# Pulse Parameters and Thresholds for (ir)Reversible Electroporation on Hepatocellular Carcinoma Cells *in Vitro*

**DOI:** 10.1177/15330338221136694

**Published:** 2023-01-04

**Authors:** K. H. K. Lindelauf, M. Baragona, M. Baumann, R. T. H. Maessen, A. Ritter

**Affiliations:** 1 Department of Diagnostic and Interventional Radiology, 39058 University Hospital RWTH Aachen, Aachen, Germany; 2 60994 Philips Research, Eindhoven, the Netherlands; 3 Institute of Applied Medical Engineering, 9165 RWTH Aachen University, Aachen, Germany

**Keywords:** hepatic cancer, HepG2, IRE, RE, ECT, minimally invasive, electric field, viability, thermal effects, cell permeabilization

## Abstract

Hepatocellular carcinoma is a leading cause of cancer-related death in many parts of the world. Traditional treatment options are not always effective. During the promising minimally invasive electroporation-based therapies, biological cell membranes are exposed to an external, sufficiently high, pulsed electric field which creates so-called nanopores into the lipid bilayer of the cell membrane. These pores can either be permanent (irreversible electroporation (IRE)), leading to apoptosis, or repairable (reversible electroporation (RE)), with continued cell function. In tumor therapy, RE is used to increase the diffusion of a chemotherapeutic drug during electrochemotherapy. For both IRE and RE, the success of the treatment is dependent on application of the appropriate electric field. Therefore, this study aims to define the pulse parameters and thresholds for IRE and RE on hepatocellular carcinoma (HepG2) cells *in-vitro*.

In a custom-made *in-vitro* setup, HepG2 cell viability (0, 5, 10, and 15 min), and the peak temperature were measured after electroporation with the different IRE and RE pulsing protocols, to determine the most successful settings for IRE and RE. A CAM/PI flow cytometric assay was performed to confirm cell permeabilization for the RE pulsing protocols with the highest cell viability.

The results indicated that an IRE pulsing protocol (70 pulses, 100 *µ*s pulse length, and 100 ms interval) with an electric field strength of 4000 V/cm was needed as threshold for almost complete cell death of HepG2 cells. A RE pulsing protocol (8 pulses, 100 *µ*s pulse length, and 1000 ms interval) with an electric field strength of 1000 V/cm was needed as threshold for viable and permeabilized HepG2 cells. The low peak temperatures (max 30.1°C for IRE, max 23.1°C for RE) within this study indicated that the reduction in HepG2 cell viability was caused by the applied electric field and was not a result of Joule heating.

## Introduction

Hepatocellular carcinoma (>80% of primary liver cancers) is a leading cause of cancer-related death in many parts of the world.^1,2^ For various tumor diseases, the liver is also a frequent location of metastases.^3,4^ For cancer patients, traditional treatment options like surgery, radiation therapy, and systemic chemotherapy are not always efficient in eradicating the tumor. Although preferred as method, resection of the primary tumor and its metastasis turned out to be only suitable for 25% of the patients in our department.^5^ This emphasizes the necessity of new treatment methods.

In this era, minimally invasive interventional cancer therapies are a popular area of research. These techniques limit the size of incisions needed resulting in less pain, decreased risk of infection, and fastened recovery of the patient.^6^ The ablation method can be either thermal (such as radiofrequency ablation (RFA) and microwave ablation (MWA), most commonly used for liver metastases) or nonthermal (such as electroporation (EP)-based therapies). Nonthermal EP-based therapies seem the most promising, mainly due to their possibility to induce apoptosis instead of necrosis, which can lead to a superior immune effect, and their avoidance of thermal side effects.^6,7^ The latter makes it possible to operate in proximity to vulnerable structures such as blood vessels, nerves, and ducts.^6,8,9^ In EP-based therapies, biological cell membranes are exposed to an external, sufficiently high, pulsed electric field (PEF), which can lead to a rapid and large increase in electric conductivity and permeability, creating so-called nanopores into the lipid bilayer of the cell membrane.^10^ When these PEF are applied to cells, two different phenomena are observed: irreversible electroporation (IRE) or reversible electroporation (RE).^10^

During the clinical treatment modality IRE, the cell membrane cannot repair the induced nanopores because of their size and amount, which causes the cell to undergo apoptosis, the natural cell death.^8–11^ At present, there are not many IRE devices on the market, so the classic NanoKnife^®^ System (AngioDynamics, New York, US), which used to be the only approved system for IRE treatment in Europe for years, remains widely used.^12^ It can be used to treat a variety of soft tissue tumors. Several applicator designs have been developed, but the system combined with straight monopolar needles remains the most popular.^13^ Hence, the need for further research and development, to improve this promising treatment.

During RE, the cells can repair their phospholipid bilayer and continue with their normal cell functions. In tumor therapy, those hydrophilic pores are used to increase the diffusion of a chemotherapeutic drug which is known as electrochemotherapy (ECT).^14,15^ Most antitumor drugs are (nearly) nonpermeant, due to their physico-chemical properties and lack of membrane transport mechanisms, which limits their antitumor effectiveness.^16^ ECT is an established therapy option in the clinic for the treatment of (sub)cutaneous tumors.^17,18^ The treatment protocol was standardized in the framework of the European Standard Operating Procedure on ECT (ESOPE), which also introduced the widely used Cliniporator™ (IGEA, Carpi, Italy) to perform ECT in clinical practice.^15,20,21,22^ More research needs to be done, to extend the application of ECT to deep-seated tumors like liver cancer.^10^

Working closely with radiologists, our department focusses on developing an improved electroporation probe, which enables the combination of IRE with ECT treatment for liver cancer.^5–23^ In both IRE and ECT, the success of the treatment is dependent on application of the appropriate electric field. The electric field can be affected by the following parameters: tissue-specific electrical properties, geometry and positions of electrodes, and electric pulse parameters.^24,25^

Therefore, this study aims to define the pulse parameters and thresholds for IRE and RE on hepatocellular carcinoma (HepG2) cells *in-vitro*.

## Methods

A translational study with several HepG2 (ATCC, Manassas, US) *in-vitro* experiments was performed to define the pulse parameters and thresholds for IRE and RE.

### Cell Culture

The human Caucasian hepatocellular carcinoma cell line HepG2 was used for all *in-vitro* experiments. The cells were subcultured by trypsinization twice a week (1:5 ratio) in Eagle's minimum essential medium (EMEM) (ATCC, Manassas, US) supplemented with 10% fetal bovine serum (FBS) (100 *µ*L/mL) and 1% penicillin/streptomycin (100 U/mL and 100 *µ*g/mL). The cells were grown in a humidified incubator at 37°C with 5% CO_2_.

### Electroporation Setup *in-Vitro*

**Figure 1.**
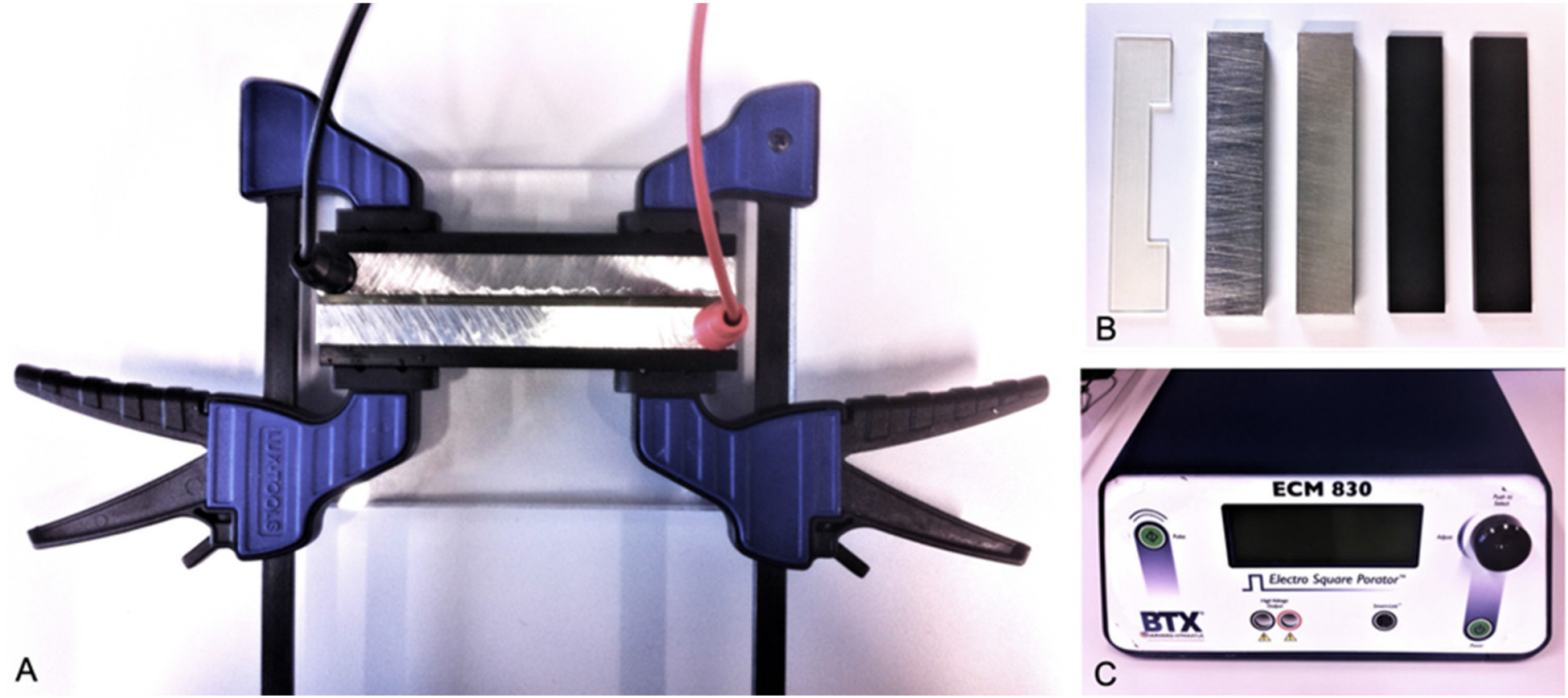
(A) Overview *in-vitro* electroporation setup. (B) Photopolymer spacer 0.2 cm (RGD720), two block electrodes (1.4571 stainless steel) and two insulators (PVC). (C) ECM 830 square wave electroporation system.

All electroporation experiments were performed *in-vitro*, using a custom-made setup designed in our department. The setup consisted of a photopolymer spacer (RGD720) with a thickness of 0.2 cm (serving as 0.7 mL cell suspension reservoir), placed between two block electrodes (1.4571 stainless steel) and PVC isolators, powered by the ECM 830 Square Wave Electroporation System (BTX, Holliston, US) (Figure 1). This setup has been described, and successfully used in several related projects over the past few years.^26^ It was made sure that the custom set-up produced a homogeneous field, and the suspension was carefully added to avoid shear stress.

### Settings for (ir)Reversible Electroporation on HepG2 Cells

First, the cell viability and heat production were measured after electroporation on HepG2 cells with different pulsing protocols, to determine the most successful pulse parameters and threshold for IRE and RE. In addition, cell permeabilization was examined to confirm pore formation for the selected RE pulsing protocols with the highest cell viability.

#### Viability

HepG2 cells were harvested, and a suspension of 1 million cells/mL was prepared in HEPES electroporation buffer (HEPES 10 mM, Sucrose 250 mM, and MgCL_2_ 1 mM). 500 *µ*L cell suspension was added to the spacer in the *in-vitro* electroporation setup.

For the IRE experiments, the following fixed parameters were used: 70 pulses, 100 *µ*s pulse length, 100 ms interval. The electric field strength was varied between 0 (control), 1000, 2000, 3000, and 4000 V/cm.

For the RE experiments, the following fixed parameters were used: 8 pulses, 100 *µ*s pulse length, 1000 ms interval. The electric field strength was varied between 0 (control), 500, 1000, 2000, and 4000 V/cm.

The number of dead and living cells, as well as the viability of the HepG2 was measured at 0, 5, 10, and 15 min after application of the different pulsing protocols. This was done using a 0.4% Trypan Blue (Gibco, Waltham, US) dye exclusion assay (1:1 ratio) on a LUNA-FL Dual Fluorescence Cell Counter (BioCat GmbH, Heidelberg, DE).

The collected data were exported to a spreadsheet for further analysis. Control groups were set to 100% viability. Accordingly, the cell viability at 0, 5, 10, and 15 min after application of the different pulsing protocols was normalized to the corresponding control group and visualized using GraphPad Prism 5 (GraphPad Software, San Diego, US). Results were displayed in graphs as mean ± standard deviation (SD). A two-way ANOVA with Bonferroni correction was performed and a two-tailed *p*-value of <0.05 was set for statistical significance (*). For both IRE and RE, the experimental test series consisted of nine independent experiments, measured in duplicates (*n* = 9). The sample size was based on preliminary data.

#### Heat Production

In accordance with the viability experiments, 500 *µ*L cell suspension (1 million HepG2 cells/mL in HEPES buffer) was added to the spacer in the *in-vitro* electroporation setup. For both the IRE and RE experiments, the same corresponding fixed and variable electroporation parameters were applied during the viability measurements described above.

While applying these different pulsing protocols to the cells, the temperature in °C was closely monitored using the Testo 882 Thermal Imaging Camera (Testo, Lenzkirch, DE). To ensure reproducibility, the thermal camera was used in combination with the Velbon Vel-flo 9 PH-368 panhead and Velbon DV-7000 tripod (Velbon Corporation, Tokyo, JP), at a 30-degree angle with a ε of 0.96. For each pulsing protocol, the peak temperature was determined, the data was exported to a spreadsheet for further analysis and visualized using GraphPad Prism 5. Results were displayed in graphs as mean ± SD. A 1-way ANOVA with Bonferroni correction was performed and a two-tailed *p*-value of <.05 was set for statistical significance (*). For both IRE and RE, the experimental test series consisted of six independent experiments (*n* = 6).

#### Cell Permeabilization

Generally, a Calcein acetoxymethyl ester (CAM)/Propidium Iodine (PI) staining is used as a life/death staining, but the combined staining of cells with these two dyes enables quantification of viability and permeability. For this flow cytometric assay, 500 *µ*L cell suspension (1 million HepG2 cells/mL in HEPES buffer), with 40 *µ*M PI (Sigma-Aldrich, St. Louis, US) was added to the spacer in the *in-vitro* electroporation setup. To confirm cell permeabilization for two selected reversible electroporation pulsing protocols, the same fixed electroporation parameters were applied as during the viability measurements described above. The electric field strength was varied between 0 (control), 500 and 1000 V/cm.

After electroporation, the cells were incubated for 5 min at room temperature and washed with FACS-buffer (1x PBS, 5% FBS, 2 mM EDTA). The cells were suspended in FACS-buffer containing 0.2* µ*M (CAM) (Sigma-Aldrich, St. Louis, US) and incubated for 10 min at RT. A BD LRSFortessa™ Cell Analyzer (BD, Franklin Lakes, US) flow cytometry device was used to process the samples. CAM signal was measured in the FITC channel, and PI signal was measured in the Texas RED channel. For each sample, 15,000 events were acquired, and the raw fluorescence data were then quantitatively analyzed and visualized with the FlowJo™ software (Ashland, US). The cells were characterized as viable and nonpermeabilized (CAM + /PI−; Q3), viable and permeabilized (CAM + /PI + ; Q2), dead (CAM−/PI + ; Q1), and unstained debris (CAM−/PI−; Q4). The test series consisted of three independent experiments, measured in duplicates (*n* = 3).

## Results

### Viability

The HepG2 cell viability was measured at 0, 5, 10, and 15 min after electroporation with the different IRE and RE pulsing protocols, as previously described.

**Figure 2.**
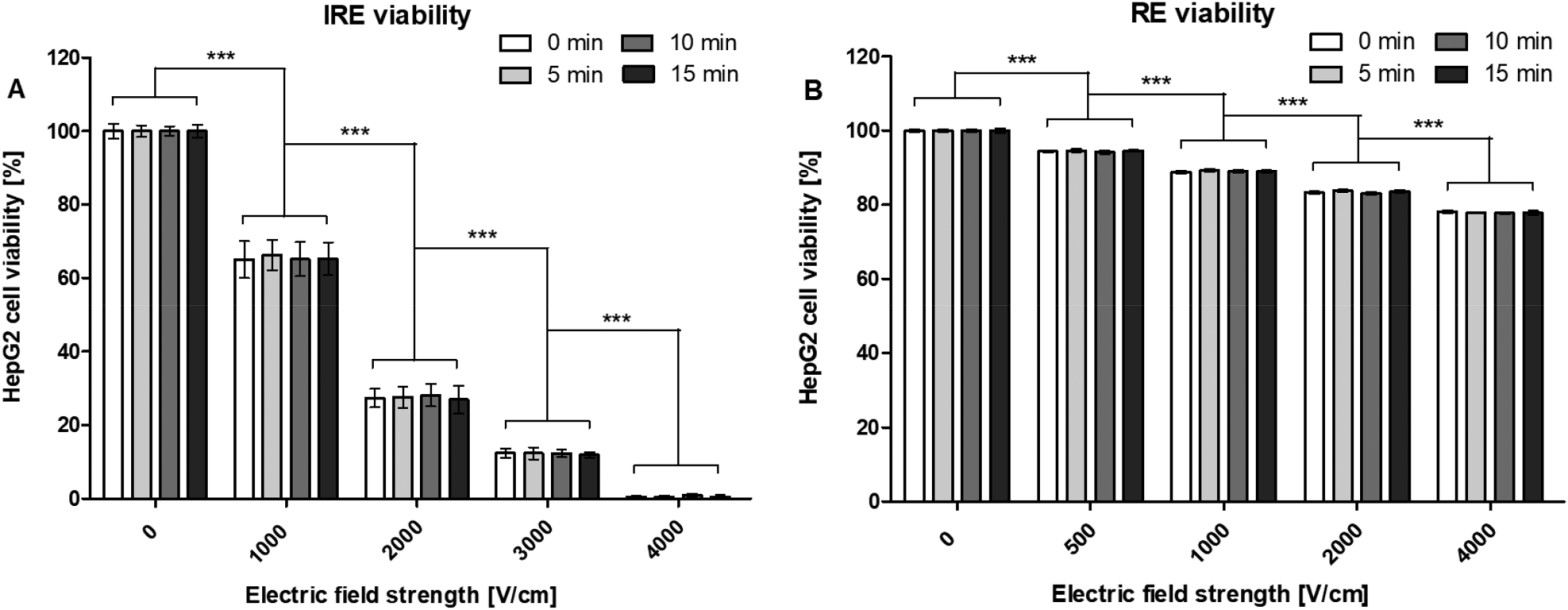
Viability (%) of HepG2 cells at 0, 5, 10, and 15 min after electroporation with the different (A) IRE and (B) RE pulsing protocols. Results are displayed as mean ± SD. A two-way ANOVA with Bonferroni correction was performed (*P < .05, **P < .01, ***P < .001). *n* = 9, in duplicates.

For the IRE experiments, the following results were found. Original cell viability of the control group was approximately 91%. Within the same pulsing protocol, no significant difference was found in cell viability between the different measuring time points. When comparing the cell viability at the same time point between different pulsing protocols a significant difference was found. The increase of electric field strength from 0 to 1000, 1000 to 2000, 2000 to 3000, and 3000 to 4000 V/cm resulted in a significant reduction of HepG2 cell viability (*P* < .001). Application of 4000 V/cm resulted in less than 1% cell viability, indicating almost complete cell death (Figure 2A).

For the RE experiments, the following results were found. Original cell viability of the control group was approximately 90%. Within the same pulsing protocol, a significant difference was found in cell viability between the different measuring time points. Values laid within 1% and standard deviations were less than 0.6%. When comparing the cell viability at the same time point between different pulsing protocols a significant difference was found. The increase of electric field strength from 0 to 500, 500 to 1000, 1000 to 2000, and 2000 to 4000 V/cm resulted in a significant reduction of HepG2 cell viability (*P* < .001). Application of 4000 V/cm caused, with approximately 78%, the lowest percentage of cell viability (Figure 2B).

### Heat Production

The heat production was measured after electroporation with the different IRE and RE pulsing protocols, as previously described.

**Figure 3.**
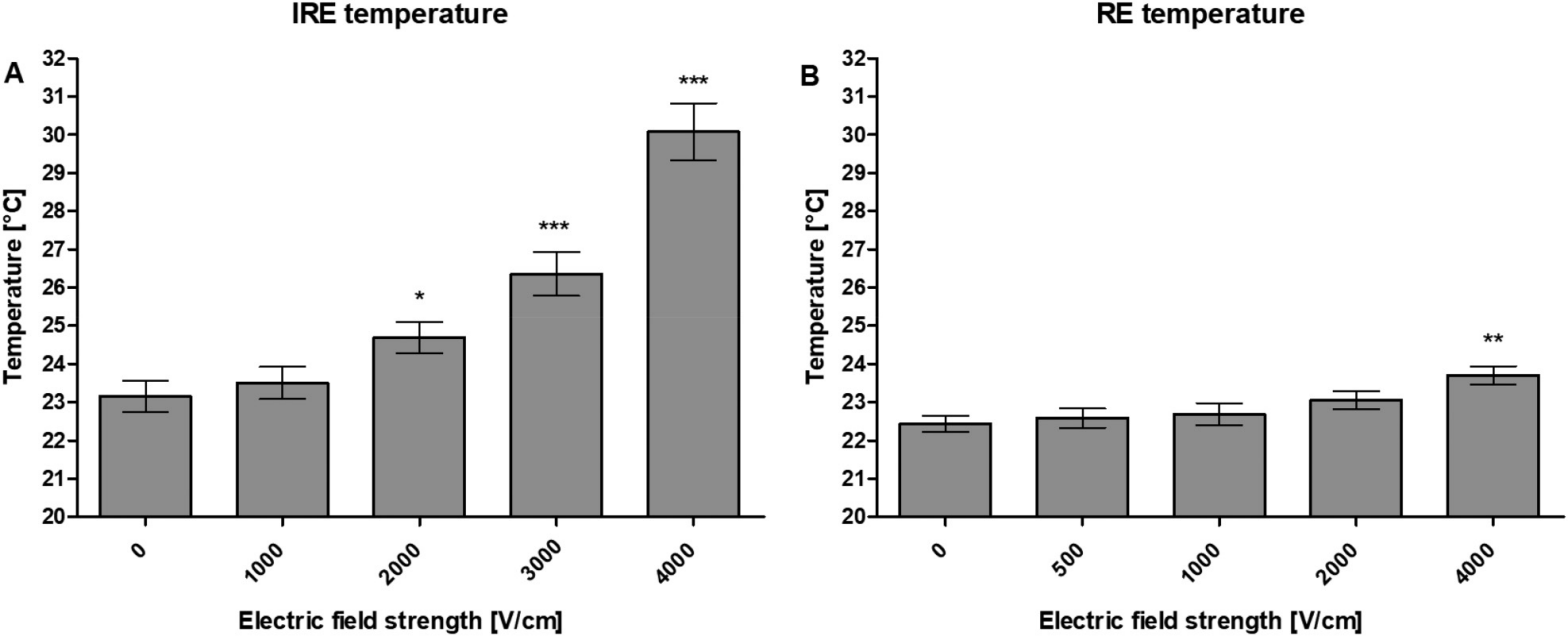
Temperature (°C) of HepG2 suspension after electroporation with the different (A) IRE and (B) RE pulsing protocols. Results are displayed as mean ± SD. A one-way ANOVA with Bonferroni correction was performed (*P < .05, **P < .01, ***P < .001). *n* = 6.

For the IRE experiments, the following results were found. The increase of electric field strength from 0 (23.2°C base temperature) to 1000 V/cm (23.5°C), did not significantly increase the temperature. The increase from 1000 to 2000 (24.7°C), 2000 to 3000 (26.4°C), and 3000 to 4000 V/cm (30.1°C) did significantly increase the temperature (*P* < .05, *P* < .001, and *P* < .001, respectively). The total temperature increase was 6.9°C (Figure 3A).

For the RE experiments, the following results were found. The increase of electric field strength from 0 (22.4°C base temperature) to 500 (22.6°C), 500 to 1000 (22.7°C), and 1000 to 2000 V/cm (23.1°C) did not significantly increase the temperature. The increase from 2000 to 4000 V/cm (23.7°C) did significantly increase the temperature (*P* < .01). The total temperature increase was 1.3°C (Figure 3B).

### Cell Permeabilization

**Figure 4.**
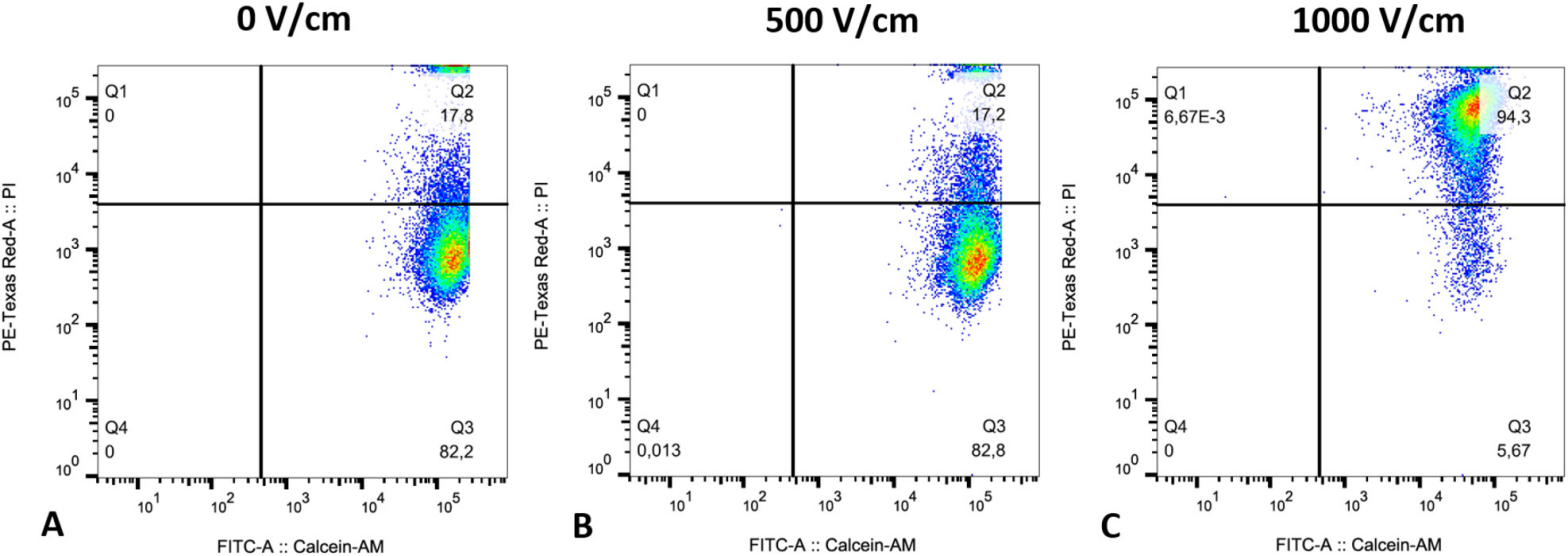
Representative dot-plot graph from CAM/pi flow cytometric assay on HepG2 cells. RE pulse protocol with an electric field strength of (A) 0 V/cm, (B) 500 V/cm, and (C) 1000 V/cm. Cells were characterized as viable and nonpermeabilized (CAM + /PI−; Q3), viable and permeabilized (CAM + /PI + ; Q2), dead (CAM−/PI + ; Q1), and unstained debris (CAM−/PI−; Q4). *n* = 3, in duplicates.

A CAM/PI flow cytometric assay was performed to confirm cell permeabilization for the RE pulsing protocols with the highest cell viability (Figure 4).

After exposure to an electric field strength of 0 V/cm (control) and 500 V/cm, respectively, 82.2% and 82.8% of the cells were characterized as viable and nonpermeabilized (CAM + /PI−; Q3), whereas 17.8% and 17.2% were characterized as viable and permeabilized (CAM + /PI + ; Q2). After exposure to an electric field strength of 1000 V/cm the majority of cells, 94.3%, were characterized as viable and permeabilized, whereas only 5.7% remained viable and nonpermeabilized. The number of dead cells (CAM−/PI + ; Q1), and unstained debris (CAM−/PI−; Q4) was neglectable in all experimental groups.

## Discussion

The human Caucasian hepatocellular carcinoma cell line HepG2 was chosen because it is a good representation of the patient population that is treated in our department and literature.^27–29^ Several *in vitro* model systems for IRE have been used in literature, including artificial membrane systems, isolated single cells, and cell suspensions.^30^ The latter is the most widely used, as it allows observation of a group of cells under external electrical stimulation, reflecting the average behavior within a cell population.^30^ In contrast to classic EP cuvettes, a custom-made cell suspension setup, designed in our department, was used. This setup had been successfully used in previous studies, and, in order to compare the results, the conditions were kept similar.^26^ Moreover, classic plastic cuvettes do not have a heat sink effect due to their low heat conductivity, whereas our setup with stainless-steel electrodes does. This is closer to reality, as the liver also has heat sink characteristics due to its vascular structure and high blood flow rate.^31^ To maximize the area of a homogeneous field and to minimize effects on the borders and corners as well as boundary effects of the electric field, it was made sure that the surface of the electroporated zone was large compared to the marginal zone. Also, the suspension reservoir was not completely filled, so the suspension was not placed on the edge of the electrodes.

It is demonstrated that electroporation buffer composition can influence cell viability.^32^ Therefore, pH changes due to pulsing were minimized by using a buffered, low-conductivity solution and stainless-steel electrodes.^33^ Although used widely in EP studies, an increase in metal ions present in stainless steel electrodes (Fe, Ni, Cr, and Mn) has recently shown to decrease cell viability in RE studies.^34^ So, ion metal release from the electrodes during pulsing should be kept minimal.

Standard IRE and RE pulsing protocols were used to compare the results with the literature and increase the translatability to the clinic. Previous studies from our department also indicated that these protocols were the most effective.^26^

The Trypan Blue dye exclusion assay was used to measure the cell viability at 0, 5, 10, and 15 min after electroporation with the different IRE and RE pulsing protocols. Incubation times were kept short, to avoid any toxicity caused by Trypan Blue. Based on the biological principle, conducting viability measurements with trypan blue after permeabilization of cells may not be optimal. However, pore closure time is completed within tens to hundreds of nanoseconds, so should not interfere with the viability measurements afterwards.^35^ This accounts for all samples, as pore closure time is practically independent of the field by which the pore was induced.^35^

Both the control groups for the IRE and RE electroporation experiments had a high and steady starting viability of 91% and 90%, respectively, which indicates a healthy cell culture needed for reliable results.

For the IRE experiments, no significant difference was found in cell viability between the different measuring time points. Within the RE experiments a significant difference was found, but given the close values, small standard deviation, and error of the technique, the difference can be disregarded. This proved that the reduction in cell viability was caused by the applied electric field and could not be attributed to external factors.

Regardless of the measuring timepoint, for both the IRE and RE experiments, the stepwise increase of electric field strength from 0 to 4000 V/cm resulted in a significant reduction of HepG2 cell viability. These results were expected, as increasing the electric field strength leads to the creation of more and bigger nanopores, which eventually lead to cell death.^18^ Although the pore formation of electric stimulated membranes is not yet completely understood, it is assumed that the electric field causes lipid molecule rearrangement on the bilayer that supports pore formation.^10,36^

For the IRE experiment, application of 4000 V/cm resulted in less than 0.5% cell viability, indicating almost complete cell death. When comparing this threshold to the data from previous studies performed in our lab with the same setup and different human pancreatic and cholangiocellular cancer cell lines, the HepG2 cells have a similar threshold to BxPc3 cells, Panc1 cells, CCLP1 cells, and SNU1079 cells, but a higher threshold than MiaPaCa2 cells (2000 V/cm).^26^ The variation in threshold for different cell lines, is in line with literature, and can be explained by differences in their size and membrane composition, as lipid differences in the outer leaflet of the cell membrane can potentially influence the level of permeabilisation.^37,38^ The variation in threshold among different types of cancer cells might be an important factor when considering the treatment planning for IRE in the clinic. Nevertheless, 637 ± 43 V/cm, a value determined by Davalos et al solely on rat liver tissue, is widely accepted as threshold for IRE in most cell types.^39^ In addition, it would be ideal if electroporation settings in vitro were directly translatable to human. But based on literature (1000–2000 V/cm) and our results (2000–4000 V/cm), the IRE thresholds for cells in suspension are higher than those measured in tissue.^30,38^ This could be explained by the compactness and spherical shape of cells in suspension, compared to their native morphology in tissue. Currently, a similar IRE pulsing protocol with a maximum of 1400 V/cm electric field strength (between the two needle electrodes) is used with the NanoKnife device for the treatment of liver cancer in the clinic.

Although IRE is classified as a nonthermal ablation method, reports have shown that Joule heating can lead to thermal damage, especially near the needles.^40^ Therefore, the peak temperature was measured during the IRE and RE experiments, with a total temperature increase of 6.9°C and 1.3°C, respectively, starting at ∼23°C base temperature. Temperature induces cell death differently; apoptosis occurs around 43°C to 50°C, whereas necrosis occurs at 50°C or higher.^41^ This means that the low-temperature changes within this study will not induce cell-death; thus, this data indicates that the reduction in HepG2 cell viability was caused by the applied electric field and was not a result of Joule heating.

For RE, induction of cell death due to the applied electric field is unwanted. The goal is to use a field that creates a majority of viable and permeabilized cells. Therefore, the RE pulsing protocols with the highest cell viability were chosen for further analysis on their pore formation. Application of a RE pulsing protocol with an electrical field strength of 0 V/cm (control) and 500 V/cm resulted in, respectively, 82.2% and 82.8% viable and nonpermeabilized cells, whereas an electric field strength of 1000 V/cm resulted in 94.3%, viable and permeabilized cells. This means that an electric field strength of 500 V/cm was not high enough to induce pore formation in the HepG2 cells, but 1000 V/cm was. This finding is also in line with the standardized treatment protocol for (sub)cutaneous tumors in the framework of ESOPE (8 rectangular pulses, 1000 V/cm, 100 µs), currently used in the clinic.^20–22^

To conclude, an IRE pulsing protocol (70 pulses, 100 *µ*s pulse length, 100 ms interval) with an electric field strength of 4000 V/cm was needed as threshold for almost complete cell death of HepG2 cells. A RE pulsing protocol (8 pulses, 100 *µ*s pulse length, and 1000 ms interval) with an electric field strength of 1000 V/cm was needed as threshold for viable and permeabilized HepG2 cells. The low peak temperatures within this study indicated that the reduction in HepG2 cell viability was caused by the applied electric field and was not a result of Joule heating.

## Abbreviations

CAM: calcein acetoxymethyl ester
ECT: electrochemotherapy
EP: electroporation
ESOPE: European Standard Operating Procedure on ECT
HCC: hepatocellular carcinoma
IRE: irreversible electroporation
MWA: microwave ablation
PEF: pulsed electric field
PI: propidium iodine
RE: reversible electroporation
RFA: radiofrequency ablation
SD: standard deviation
